# A Question of Legitimacy

**Published:** 1845-05

**Authors:** 


### A QUESTION OF LEGITIMACY.

Our friend, Professor Cobb, has lately been consulted respecting a case involving a question of paternity, which came up not long since in one of the courts of a Southern State. The facts stated are as follows: A white woman, the wife of a planter of wealth and respectable connexions, gave birth to a male child of so dark a complexion that unpleasant suspicions were awakened among her acquaintances. Her husband died subsequent to the birth of the child, and after remaining a widow four or five years she again married. A doctor, it seems, in an evil hour, charged her with incontinence, alleging that "she had given birth to a mulatto child." Upon this an action for slander has been brought, and is still pending, the jury having failed, on the first trial, to agree on a verdict. Nine doctors appeared as witnesses, who expressed opinions widely variant touching the merits of the case.

It was proved that the first husband, the alleged father of the boy, was a man of fair complexion, being of German extraction, but that his mother and two of his uncles were dark like the child, and that his family in Germany were descended from the Gipsies. It was further stated in the trial, that during the pregnancy of the mother with this child she was repeatedly frightened by reports of "negro insurrections."

The appearance of the boy is described as remarkable. His surface presents different shades of color, the chest and axilla being nearly white, while the abdomen is dark, the change occurring abruptly and being marked by a well defined line. The glans penis "is quite blue," while the other parts of the genital organs are of the complexion of the general surface. The boy has been growing gradually whiter since birth; "his hair is nearly straight—a little curled, but not kinked;" his feet and ankles present nothing of the negro peculiarity; his whole appearance might suggest the thought that he was the "product of a white woman and a mulatto man." Such is the description of the boy's physiognomy.

It was proved by the defendant that the character of the plaintiff had not been above suspicion, and that criminal connexion with her carriage-driver had been possible, if not probable.

Some of the questions upon which the medical witnesses differ are thus put by Professor Cobb's correspondent:

"Is not a mulatto from a white woman darker than one from a black woman?

Are there any anatomical or physiological signs by which negro blood might with certainty be detected?

Is it not usual for mulattoes to grow darker instead of whiter?

Would you place any reliance upon the color of the glans penis?

Do you think it possible that a *nævus materni* could cover nearly the whole body?

Do you think it possible for color to show itself in the third and fourth generation?

Do you know of any mode of bleaching by which the skin might be rendered white?"

The narrator is one of the nine medical witnesses in this suit, and is evidently very much perplexed. He says, "it has been my misfortune since I commenced the practice of physic to be called upon to give evidence in many cases of medical jurisprudence, but never before in one so difficult as this."

The gallantry of our distinguished colleague, no doubt, will tempt him to lean to the side of the lady and the legitimacy of her offspring, and the following facts in the case will support him in that opinion: namely, that the boy has been growing whiter since his infancy; that he is wanting in the characteristics of the negro race about the heel and ankle; that he is descended of the Gipsies in whom the dark complexion is a hereditary quality; and that some of his ancestors were as black as himself. Still it must be admitted that these proofs are not conclusive of the purity of blood, and the question is one which it may be impossible to place beyond the reach of controversy. Nature in her operations in this obscure walk seems not to be governed by very settled laws. Thus we are told of an English woman married to a black man, of whom the offspring was quite black; and of a similar case in which the child resembled the mother in fairness of features, the whole skin being white, "except some spots on the thigh which were as black as the father." White, in his work on the Gradation of Man, mentions a more remarkable case—that of a negress who had twins by an Englishman, one being perfectly black, with short, woolly, curled hair, the other white with hair resembling that of a European. Beck, in his Medical

Jurisprudence, has more cases going to show the difficulty of establishing any universal rule on the subject. Parental likeness in the estimation of Lord Mansfield is one of the strongest arguments in favor of legitimacy. In a case which came before him involving this question he said, "I have always considered likeness as an argument of a child being the son of a parent, and the rather as the distinction between individuals in the human species is more discernible than between other animals. A man may survey ten thousand people before he sees two faces exactly alike, and in an army of ten thousand men every man may be known from another. If there should be a likeness of feature, there may be a difference in the voice, gesture or other characters; whereas a family likeness runs generally through all of these; for in everything there is a resemblance as of feature, voice, attitude and action." This test, we should suppose, might avail in the case under consideration.

But Lawrence in his Lectures cites many facts going to show that the above law of transmitted likeness is not without exceptions. He remarks: "Children do not always resemble their parents; and hence we have occasionally persons produced in each race with characters approaching those of the other races. Among the white races of Europe scattered instances of individuals with skins nearly as dark as those of the Mongols or South-Sea Islanders are not unfrequent. I lately saw a girl," he continues, "whose dark-olive skin and jet-black hair, very much like those of a Chinese, joined to English features, made me suppose that there was some mixture of blood: it turned out, however, that her parents were both English: the mother dark, but not of so deep a tint as the daughter, and the father fair."

Our memory, happily, furnishes us with but one solitary case in which children were born of a white woman by a black man, and in this the offspring were rather bright mulattoes, partaking in the usual degree of the characters of the two races. Such instances, we are convinced, are of rare occurrence; and we hope that the lady involved in the vexatious suit to which we referred at the beginning of this article will be able to satisfy the jury, and society, that her unlucky boy inherits his dark skin from ancestors at least as respectable as the Gipsies. Y.

